# Prevalence and Risk Factors Associated with *Theileria annulata* Infection in Two Bovine Portuguese Autochthonous Breeds

**DOI:** 10.3390/pathogens12050669

**Published:** 2023-05-02

**Authors:** Diana Valente, Ana Paula Dutra, Nuno Carolino, Jacinto Gomes, Ana Cláudia Coelho, Pedro Espadinha, José Pais, Inês Carolino

**Affiliations:** 1 CIVG—Vasco da Gama Research Center, EUVG—Vasco da Gama University School, 3020-210 Coimbra, Portugal; 2 Escola de Ciências Agrárias e Veterinárias, Universidade de Trás-os-Montes e Alto Douro, Quinta de Prados, 5000-801 Vila Real, Portugal; 3 CECAV—Animal and Veterinary Research Center, Universidade de Trás-os-Montes e Alto Douro, Quinta de Prados, 5000-801 Vila Real, Portugal; 4 Unidade Estratégica de Investigação e Serviços—Produção e Saúde Animal (UEIS-PSA), Instituto Nacional de Investigação Agrária e Veterinária, Av. Da República, Quinta do Marquês (edifício sede), 2780-157 Oeiras, Portugal; 5 Polo de Inovação da Fonte Boa—Estação Zootécnica Nacional, Instituto Nacional de Investigação Agrária e Veterinária, 2005-424 Santarém, Portugal; 6 CIISA—Centre for Interdisciplinary Research in Animal Health, Faculty of Veterinary Medicine, University of Lisbon, 1300-477 Lisbon, Portugal; 7 Associate Laboratory for Animal and Veterinary Sciences (AL4AnimalS), Faculty of Veterinary Medicine, University of Lisbon, 1300-477 Lisbon, Portugal; 8 Escola Superior Agrária de Elvas, Instituto Politécnico de Portalegre, 7350-092 Elvas, Portugal; 9 Associação de Criadores de Bovinos da Raça Alentejana, Herdade da Coutada Real—Assumar, 7450-051 Assumar, Portugal; 10 Associação de Criadores de Bovinos Mertolengos, 7006-806 Évora, Portugal; 11 ISA—Instituto Superior de Agronomia, Universidade de Lisboa, 1349-017 Lisboa, Portugal

**Keywords:** Tropical Bovine Theileriosis, tick borne diseases, indigenous Portuguese breeds, epidemiology, control strategies

## Abstract

Tropical Bovine Theileriosis is an important tick-borne disease. This study aims to assess the occurrence of *Theileria annulata* infection in two indigenous Portuguese cattle breeds. A total of 843 blood samples collected from animals of Alentejana (n = 420) and Mertolenga (n = 423) breeds were analyzed. The detection of *Theileria annulata* was determined by amplification of a fragment of the merozoite-pyroplasm surface antigen gene with 319 base pairs (bp). The prevalence found (10.8%) is lower than that reported in previous studies (21.3%). A statistically significant difference was found for positivity between breeds (*p* < 0.05). There is also a higher probability of older animals being positive compared to younger ones (*p* < 0.05). The region where Mertolenga animals are located is shown to have a significant impact on positivity (*p* < 0.05). Thus, the development of sustainable *T. annulata* control strategies and their implementation, adapted to the epidemiological conditions of higher risk, will be extremely important.

## 1. Introduction

*Theileria annulata* is a tick-borne hemoprotozoan parasite responsible for Bovine Tropical Theileriosis, hereafter named “Theileriosis”, a tick-borne disease (TBD) that is globally distributed and prevalent in Asia, Middle East, Southern Europe and Northern Africa. Based on its geographical location, it is also called Mediterranean Theileriosis. Tropical Bovine Teileriosis threatens about 250 million cattle worldwide, having a very important negative impact on animal production, especially in developing countries [1]. Thus, this disease promotes increased mortality rates on the farm, reduces production and limits the development programs of different breeds [2,3].

The presence of a competent tick vector species is the main factor determining *Theileria annulata* circulation in a certain area [4]. *T. annulata* is transmitted by ticks that may fall to the ground three times in each of its life cycle stages, namely larva, nymph and adult (three-host ticks) or only two, with the larva and nymph being in one host and the adult in another (two-host ticks) [5]. The transmission of this haemoprotozoan parasite by ticks of the genus *Hyalomma* (*H. marginatum, H. anatolicum, H. lusitanicum, H. scupense* and *H. dromedarri*) is widely described [2,3,6,7]. Despite this, *Theileria annulata* has also been detected in ticks of the species *Rhipicephalus bursa*, and there is a possibility of transmission by other species of the genus *Rhipicephalus* (*R. evertsi evertsi*, *R. decoloratus* and *R. annulatus*) and *Amblyomma* (*A. variegatum* and *A. lepidum*) [8,9]. Several species of ticks have been described in Portugal, among which four are known to be competent in the transmission of *T. annulata*, namely *H. lusitanicum*, *H. marginatum*, *R. bursa* and *R. annulatus* [10,11]. The life cycle of *Theileria annulata* includes different morphological phases that occur in two hosts: an invertebrate host (tick) and a vertebrate host (cattle) [12,13]. Schizogony and merogony occur in cattle, while the zygote and kinete forms arise in ticks. Ticks are infected by ingesting erythrocytes with the piroplasm and the sporozoites are then transmitted to the bovine along with the saliva during meals by adult ticks and ticks in the nymph stage. After, the merozoites infect the erythrocytes and are known as piroplasms because they trigger the clinical sign of fever in the host animal [13].

Among the conventional breeding species, *Bos taurus* ones are more susceptible to Theileriosis, which cause a serious inflammatory process, with high mortality rates. On the other hand, in *Bos indicus* breeds, such as Sahiwal, which inhabit endemic regions, pro-inflammatory cytokines dependent on the acute phase response are controlled and survival rates are higher. In the initial phase of sporozoite invasion, antibodies produced against epitopes on its surface may assist in reducing infection, and the role of the humoral response in *T. annulata* infection is very important [8]. It is estimated that the antibodies produced prevail for at least six months [14,15]. In addition, T cells, NK cells and their products also contribute to immune protection [8]. Theileriosis diagnosis can be based on traditional methods, such as the evaluation of the presence of clinical signs (hyperthermia, anemia and jaundice) and post-mortem findings, blood smears and serology, but also on molecular methods, with greater sensitivity and specificity [3,16,17,18]. Control of theileriosis is commonly based on treatment of infected animals, application of acaricides and vaccination [19]. A new alternative control strategy for Theileriosis could be the use of resistant breeds. This is especially important for endemic regions, such as southern Europe [20]. This strategy is already being studied, for example, for the Sahiwal breed, which has been identified as a breed resistant to ticks and infection by this agent [21,22].

In Europe, there are some studies that have determined the prevalence of *T. annulata*, namely in Southern Europe, where it is more common [3]. Thus, in 1996, in Macedonia (Greece), a prevalence of 2% was found, using the indirect fluorescence antibody (IFA) technique [23]. In 2017, a prevalence of 22.4% was found in Madrid (Spain), using polymerase chain reaction (PCR) [24]. In Sicily (Italy), in 2021, a prevalence of 26% was found, using IFA [25]. In Portugal, several studies have been carried out to determine the prevalence of the Theileriosis. In 2008, Antunes studied its prevalence on a farm in the Ribatejo region, by blood smears stained with Giemsa, and found that 27% of the animals were infected by *Theileria* spp. [26]. The following year, in the Alentejo, a frequency of 46.2% of infected cattle was estimated using IFA [27]. In 2013, Gomes et al. determined the prevalence of *T. annulata* in different regions of Portugal, with reverse line blotting (RBL), finding a prevalence of 3.3% in the North, 12.4% in Center, 33.5% in Lisboa e Vale do Tejo, 29.2% in Alentejo and 15.6% in Algarve. At the national level this frequency was 21.3% [28]. Little is currently known about the impact of the disease in Portugal, but there are reports of the lethality of this disease in calves under four months of age [29]. The present study was performed to evaluate the epidemiological status of *T. annulata* infection in Portugal, in two indigenous Portuguese cattle breeds, namely the Alentejana and the Mertolenga breed, and the risk factors for infection. The aim is to analyze the influence of factors such as the breed, sex and age of the animal, the district where the farm is located, the months in which the sample was collected, the size of the farm, the presence of dogs or other animals on the farm and the use of parasiticides on *T. annulata* positivity. The identification of possible risk factors in asymptomatic carrier animals provides evidence-based guidance for effective control measures.

## 2. Materials and Methods

### 2.1. Study Areas and Subjects

**Figure 1:**
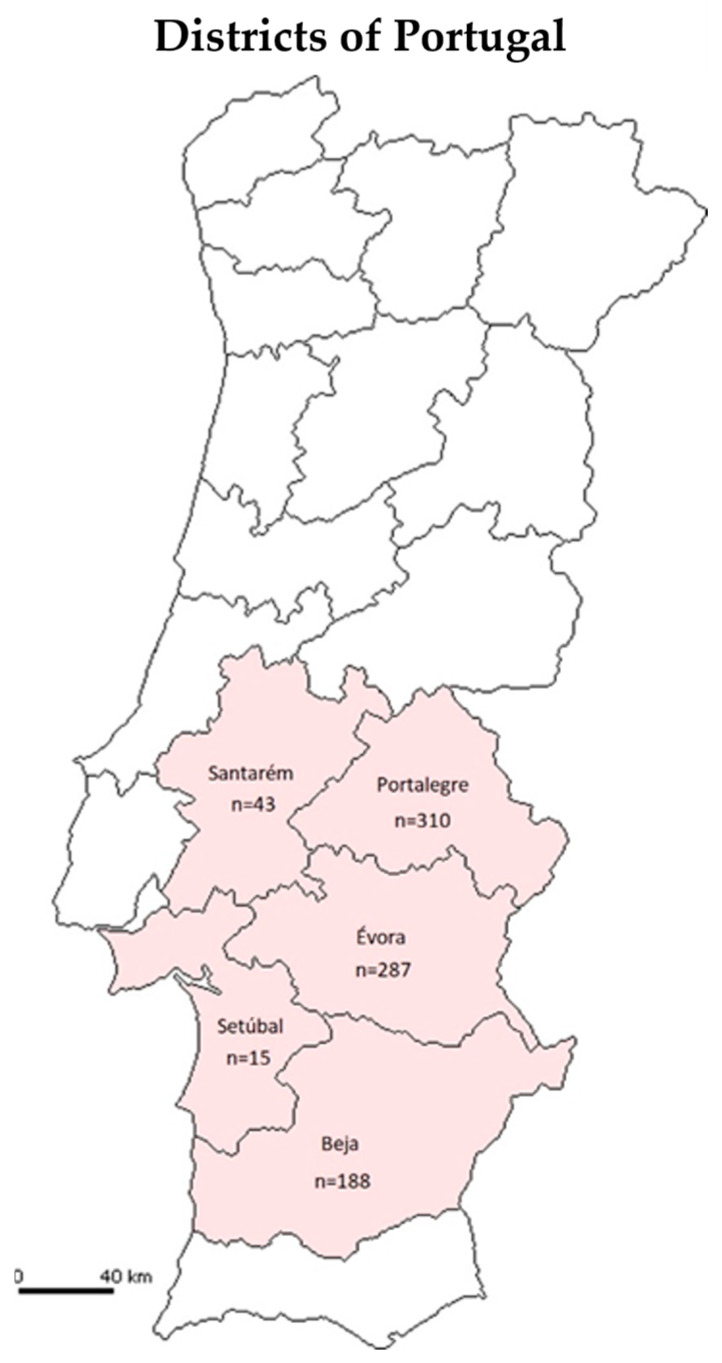
Distribution of the samples collected in 2018 and 2019, by the different districts of the Alentejo.

The blood samples used in this study were collected in the Alentejo region of Portugal (Figure 1). This is the place of origin of the two autochthonous Portuguese breeds (Alentejana e Mertolenga) under study and an endemic region of *T. annulata*. In general, the climate of this region is a temperate climate with rainy winters and dry and hot summers (Csa) (Köppen Climate Classification). The temperature of this region can oscillate on average between 33.5 °C (average maximum temperature of the hottest month—August) and 4.3 °C (average minimum temperature of the coldest month—January) [30].

All species of ticks previously described in Portugal, in which *T. annulata* has already been detected, namely *H. lusitanicum*, *H. marginatum*, *R. bursa* and *R. annulatus*, were found concentrated in the south of Portugal, the region where our study was carried out. This will be a consequence of the existence of higher temperatures, large forests of *Quercus* spp. associated with large areas of spontaneous grasses, and with a large presence of grazing ruminants [31]. Furthermore, there are reports that, for example, the species *H. lusitanicum* is mainly distributed along climate Csa, while *H. marginatum* is found in several climates, in which the Csa is included [32].

### 2.2. Sample Collection

A total of 843 blood samples were randomly collected from cattle without clinical signs of infection, from the Alentejo region between November 2018 and December 2019. These animals belonged to two distinct native Portuguese breeds: Alentejana (n = 420) and Mertolenga (n = 423). These animals all belong to farms with extensive or semi-extensive farming systems, which means that they are animals which are outdoors and grazing. Approximately 3 to 5 mL of blood was collected from the jugular vein of each animal and stored individually in tubes with EDTA (ethylenediaminetetraacetic acid). The blood collection was carried out by the technical of the Autochthonous Cattle Breed Associations under study. The tubes with the blood sample were frozen (−20 °C) before being sent to the laboratory. During transport and storage, the temperature conditions were maintained.

At the time of the sample collection, a questionnaire was applied, allowing for obtaining information such as breed, sex, age of the animal, district where the farm is located and corresponding territorial units.

### 2.3. Molecular Testing and Sequencing

The blood was subsequently used to extract DNA using the Cytogene^®^Blood Kit (India, Cytogene), following the manufacturer’s instructions.

For PCR amplification of a fragment of a *T. annulata* merozoite-piroplasm surface antigen gene, Tams 1 gene, a set of primers was used (F 5′ CAA ATT CGA GAC CTA CGA TG 3′ and R 5′ CCA CTT (A/G) TC GTC CTT AAG CTC G 3′), allowing for the amplification of a fragment with about 319 base pairs (bp), as described by Santos et al. [33]. The reaction mixture was prepared in a final volume of 25 μL, consisting of 5 μL Multiplex PCR Mix (5x HOT FIREPol^®^ MultiPlex Mix Ready to Load, Solis BioDyne, ref: 04-36-00120), 1.25 μL forward and reverse primer *T. annulata* [10 Mm], 12.5 μL sterile distilled water and 5 μL of extracted DNA sample. PCR conditions included an initial pre-denaturation phase for 15 min at 95 °C, followed by 32 cycles of denaturation at 95 °C for 30 s, primer annealing at 55 °C for 30 s and extension at 72 °C for 30 s. A final extension was performed at 72 °C for 7 min. After this it is kept on standby at 4 °C. A positive *T. annulata* sample and a negative sample were amplified during each of the PCR reactions performed as positive and negative controls, respectively. The positive control samples are the property of the Parasitology Laboratory of the National Institute for Agricultural and Veterinary Research (INIAV) and the negative control samples resulted from a mixing reaction without DNA but with sterile distilled water of the same volume, and these samples were subjected to the same reaction conditions as all the others.

Amplified samples were analyzed by electrophoresis in 1.5% agarose gel. Positive and negative PCR controls were run with each series of amplifications, and on each gel, a molecular weight marker was placed (NZYDNA VI). The gel was visualized with an ultraviolet (UV) transilluminator.

Results were obtained for all 843 animals sampled, and some representatives of those positive for *T. annulata* were validated by sequencing the amplified DNA fragment.

### 2.4. Statistical Analysis

Statistical analysis was performed using SAS^®^ Version 9.4 software (SAS Institute Inc., Cary, NC, USA, 2019). Statistics descriptive for several factors and variables evaluated were calculated based on the MEANS and FREQ procedures. The variables under study included positivity for *T. annulata* infection, breed, sex, age, farm district, season of the year (spring, summer, autumn or winter) and the month of the year in which the samples were collected, using a division between hot months (May, June, July, August, September and October) and cold months (January, February, March, April, November and December), the Territorial Units Used for Statistics (NUTS III—Alto Alentejo, Baixo Alentejo, Alentejo Central and Lezíria do Tejo), the number of animals on the farm (Class 1—less than 100 animals; Class 2—value equal to or greater than 100 animals and less than 500; Class 3—value equal to or greater than 500 animals), the presence of dogs or other animals on the farm and the use of parasiticides (active substance used) [30]. After that, univariate logistic regression analysis was performed to assess the main factors associated with *T. annulata* positivity, using PROC LOGISTIC, and considering the data of all test animals together. Factors with significance levels greater than 5% (*p* > 0.05) were excluded. Later, the same statistical tools were applied to analyze the animals’ data, considering each breed individually.

## 3. Results

### 3.1. Prevalence of Theileria annulata in Cattle Blood Samples

During the present investigation, 7.1% (30/420) of blood samples from the Alentejana breed and 14.4% (61/423) of blood samples from the Mertolenga breed were positive. Thus, the average prevalence of infected animals in this study was 10.8% (91/843).

In this research work, it was also found that there was a higher prevalence of *T. annulata* positive females than males. Considering the age, in the case of males, the highest prevalence is between 1 and 3 years and in the case of females, positivity is more prevalent in animals older than 3 years (Table 1).

As for age, it was found that the percentage of positive animals increases with age. Thus, in animals aged 3 years or more, a positivity rate of 12.9% (11/85) in the Alentejana breed and 27.0% (10/37) in the Mertolenga breed was determined, while in animals aged between 1 and 3 years, only 6.6% (18/274) in the Alentejana breed and 19.5% (51/262) in the Mertolenga breed were positive. Furthermore, in younger animals, less than 1 year old, positivity is very low (1.6% (1/61) in the Alentejana breed and 0.0% (0/124) in the Mertolenga breed).

Considering the district and the age of the animals, we find that the only district presenting positive animals under 1 year old is Évora. On the other hand, between 1 and 3 years old, the district with the highest prevalence is Beja, followed by Évora and we find the lowest prevalence for this age range in Setúbal. As for animals aged 3 years or more, the highest prevalence is found in Santarém, followed by Portalegre, and the district with the lowest prevalence is Beja (Table 2).

Analyzing other epidemiological factors, we found that the highest percentage of positive animals of the Alentejana breed is found in Baixo Alentejo, followed by Central Alentejo. The same is true for animals of the Mertolenga breed, where the highest prevalence is found in Baixo Alentejo, followed by Central Alentejo. Regarding the month of sampling, we verified that in the Alentejana breed, the highest percentage of positive animals is obtained when the blood sampling is done during hot months, contrary to what happens with the animals of the Mertolenga breed. In the second case, the highest percentage of positive animals was obtained when the sample is taken during cold months. When we consider the season of the year when the sampling is done, we verify, in the case of the Alentejana breed, that the highest percentage of positive animals is obtained during summer, followed by spring, which does not happen in the case of the Mertolenga breed animals (the highest percentage of positive animals is found during winter, followed by autumn). Furthermore, it can be stated that for animals of both breeds, there is a higher prevalence of positive animals on smaller farms (less than 100 animals). Regarding the presence of dogs on the farm, only in animals of the breed Alentejana is the prevalence of positive animals higher in farms with dogs. In animals belonging to farms of both Mertolenga and Alentejana cattle breeds, the prevalence of positive animals is higher on farms with no other animals. Finally, regarding the use of ectoparasiticides, in the case of the Alentejana breed, we found a higher prevalence of positive animals on farms using ivermectin and a lower prevalence on farms using deltamethrin. On the other hand, in animals of the Mertolenga breed, the highest prevalence of positive animals is found in animals belonging to farms using moxidectin and cypermethrin and the lowest prevalence is in animals belonging to farms using ivermectin, deltamethrin and moxidectin (Table 3).

### 3.2. Analysis of Epidemiological Factors

**Figure 2:**
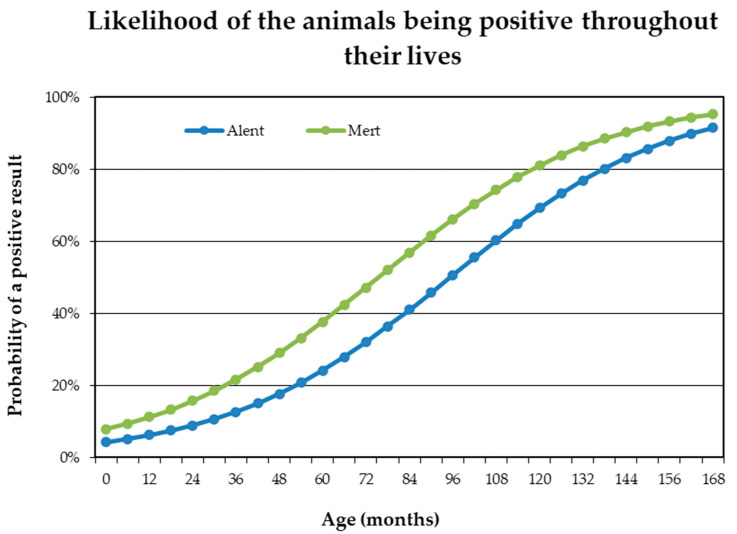
Evolution of the probability of an animal being positive with age for the Alentejana (Alent) and Mertolenga (Mert) breeds.

The probability of the animal being positive was studied based on different criteria using logistic regression. Considering all animals under study and only the breed variable, an odd ratio of 3.611 (*p* < 0.05) was obtained. Thus, we could state that the probability of a Mertolenga animal being positive for *T. annulata* would be 3.611 times higher than that of an Alentejana animal. However, in the next step, we also decided to include the following factors: breed and age (as a covariate) and district and age (as a covariate). Using these models, sex was found not to significantly influence positivity for *T. annulata* (*p* > 0.05). Nevertheless, it was found that with increasing age, animals are more likely to be positive for Theileriosis in both breeds (Figure 2). Thus, based on the Logarithm of the probabilities, we can say that for each unit of increase in age (month), the logarithm varies (increases) by 0.0326 (*p* < 0,05). This means that for a one month increase in age, the odd ratio of the probability of a positive result varies by approximately 3.3% (Figure 2).

**Figure 3:**
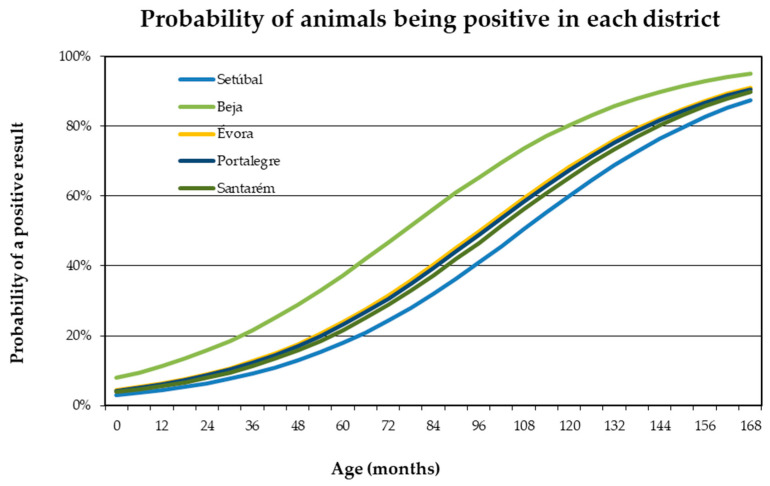
Evolution of the probability of an animal being positive, considering its age and the district of the farm to which it belongs.

On the other hand, although the district does not have a statistically significant influence on positivity to *T. annulata*, when analyzing the data of all animals (of both breeds) simultaneously (*p* > 0.05), we found that the probability of an animal being positive increases with age in all districts under study (Figure 3).

All the other variables under study were also analyzed, considering all the animals, and in no case was there a statistically significant influence on positivity for *T. annulata*. Therefore, it was decided to implement this analysis in the animals of the two breeds under study (Mertolenga and Alentejana) separately. In the case of the Alentejo breed, there was no statistically significant effect of any of the variables. As for animals of the Mertolenga breed, when analyzed separately, a statistically significant relationship was found between positivity and NUTS III (*p* = 0.0245). In this analysis, the following groups were considered: Lezíria do Tejo and Alto Alentejo, as opposed to Alentejo Central and Baixo Alentejo. It was then verified that the probability of finding a positive animal of the breed Mertolenga in Central Alentejo and Baixo Alentejo is 2.341 times greater than the probability of finding a positive animal of this breed in Lezíria do Tejo and Alto Alentejo (OR = 2.341; IC = 95%). It was not possible to establish any other statistically positive relation between the epidemiological factors studied.

## 4. Discussion

Tick-borne pathogens, such as *T. annulata*, have a huge economic and welfare impact, contributing to important mortality rates and loss of productivity. As previously mentioned, in 2013, a prevalence of *T. annulata* in cattle of 29.2% was found by RBL in the Alentejo region, the dominant area of our study [28]. Although there are some reports of a higher sensitivity of RBL compared to PCR, there is also an indication that the results obtained by RBL should be interpreted with greater caution, because the fact that we identify a very small amount of the parasite genome, does not mean that the animal is a carrier [35,36,37]. Thus, we found that, in our study, the prevalence is lower than both the national prevalence found in 2013 and the prevalence in Alentejo in the same year. This would not be the expected result. With increasing temperature, resulting from climate change, the spread of tick-borne diseases is expected to increase [38,39]. Thus, one of the reasons that may justify the low prevalence in this study may be the greater resistance of the animals evaluated, which are animals of indigenous Portuguese breeds (Mertolenga and Alentejana). There are several studies indicating a significant difference in resistance of different animal breeds, such as the Sahiwal breed (*Bos indicus*) and the Holstein breed (*Bos taurus*) [21,22,40].

Analyzing the different epidemiological factors, in this study, we recorded, for both breeds, a higher prevalence of females infected with *T. annulata* (11.5%), although there is no statistically significant relationship between positivity for *T. annulata* and sex. This may be associated with the fact that we had a much higher number of females (764), when compared to the number of males (69). In addition, there may also be a relation with age distribution. In this study we found that the probability of finding positive animals increases significantly with age (each month the probability increases by 3.3%) We also know that the proportion of younger animals (up to 1 year) is higher in the males under study (64.6%; 51/79). In the case of females, we found fewer animals in this age group (17.5%; 134/764). Moreover, the oldest male is 4 years and 7 months old while the oldest female is 14 years and 2 months old. On the other hand, there are already some studies indicating a higher prevalence of females positive for piroplamosis, compared to males [41,42,43,44]. This may be the result of increased hormonal stress associated with childbirth and milk production [42,43]. Furthermore, this is also in line with the information provided by Kamani et al. (2010) [45] and by Parveen et al. (2021) [46] that indicate a higher prevalence in females because they are kept longer for different purposes, such as reproduction and milk production, but also because they may not receive adequate feed to meet their high nutritional demand, associated with their productive function. Regarding age, we know that in many infectious and parasitic diseases there is a lower exposure of young animals, which will naturally increase with age [24]. There are already some references indicating a higher prevalence of *T. annulata* infection in older animals, compared to younger ones [30,34,47,48]. Similar to our study, Flach et al. also reported a statistically significant influence of age on the subclinical infection of cattle, being more likely to find older animals positive for *T. annulata* and without clinical signs of Theileriosis [49].

Other epidemiological factors such as the district, the season and month of sampling (hot or cold), the number of animals on the farm, the presence of dogs or other animals and the ectoparasiticide used did not have a statistically significant influence on positivity for Tropical Theileriosis. The districts under study are very similar as regards their climatic conditions. Despite this, in animals of the Mertolenga breed, there was an influence of the NUTS III groups where the farm is located. In the area corresponding to Baixo Alentejo and Central Alentejo, there is a greater probability of finding animals positive for *T. annulata* (2.341 times greater), than in the area corresponding to Alto Alentejo and Lezíria do Tejo. Considering the geographical proximity of each of the regions included in the two groups, with the Lezíria do Tejo and Alto Alentejo being closer to the Centre of Portugal and the Central Alentejo and Baixo Alentejo further South, the difference found may possibly be justified by the existing climatic differences in Portugal [50]. There are some studies on several species of ticks that indicate that the pre-oviposition period and the oviposition period are faster in environments with higher temperatures, as well as the number of eggs produced and their hatchability [51,52]. Thus, temperature differences between regions may be responsible for the difference in tick prevalence and, consequently, positivity of cattle for tick-borne agents. Further studies are needed to evaluate these hypotheses.

Finally, although it was hypothesized that the size of the farm, the presence of dogs or other animals on the farm and the use of ectoparasiticides had an influence on the positivity of the animals, this was not found to be the case. Although there are different farms in terms of size, they all use similar production regimes, where all animals have access to the outdoors (pasture). Regarding the presence of dogs and other animals on the farm, we also verified the inexistence of a significant relation. There are some studies that prove the infection of dogs by *T. annulata* and hypothesize that these are considered natural carriers or reservoirs, which may contribute to the infection of cattle and the presence of the agent in this type of farm [53,54,55,56,57]. In this study we were unable to prove this relation, and further research is needed in this regard.

## 5. Conclusions

In conclusion, we report a lower prevalence of *T. annulata* infection in cattle compared to previous studies. This may be related to the breeds under study, indigenous Portuguese breeds, which may be more resistant. A higher risk of infection was found in older animals compared to younger ones. There was also a relationship between infection and climatic conditions, namely temperature. The data produced by this study highlights the importance of prophylactic detection and should be considered in the development of control strategies for Tropical Bovine Theileriosis, with the aim of improving the health and welfare of animals and the productivity of livestock farms.

## Figures and Tables

**Table 1:** Comparison of the prevalence of *T. annulata* in blood samples from female and male cattle, considering age. % prevalence is shown in parentheses.

Sex	Age	Number of Samples	*T. annulata* Positive Samples (%)	*T. annulata* Negative Samples (%)
**Male**	≤1 year	51	0 (0.0)	51 (100.0)
	>1 and <3 years	19	3 (15.8)	16 (84.2)
	≥3 years	9	0 (0.0)	9 (100.0)
	**Total**	79	3 (3.8)	76 (96.2)
**Female**	≤1 year	134	1 (0.7)	133 (99.3)
	>1 and <3 years	517	66 (12.8)	451 (87.2)
	≥3 years	113	21 (18.6)	92 (81.4)
	**Total**	764	88 (11.5)	676 (88.5)
**Total**		843	91 (10.8)	752 (89.2)

**Table 2:** *T. annulata* prevalence in animals belonging to different age groups, considering the various districts of Alentejo under analysis, for both breeds. The age groups were adapted from previous studies [34]. % prevalence is shown in parentheses.

		≤1 Year			>1 and <3 Years			≥3 Years		
District	Breed	Number of Samples	*T. annulata* Positive Samples (%)	*T. annulata* Negative Samples (%)	Number of Samples	*T. annulata* Positive Samples (%)	*T. annulata* Negative Samples (%)	Number of Samples	*T. annulata* Positive Samples (%)	*T. annulata* Negative Samples (%)
**Beja**	Alentejana	9	0 (0.0)	9 (100.0)	44	7 (15.9)	37 (84.1)	14	0 (0.0)	14 (100.0)
	Mertolenga	22	0 (0.0)	22 (100.0)	98	21 (21.4)	77 (78.6)	1	1 (100.0)	0 (0.0)
	**Total**	31	0 (0.0)	31 (100.0)	142	28 (19.7)	114 (80.3)	15	1 (6.7)	14 (93.3)
**Évora**	Alentejana	24	1 (4.2)	23 (95.8)	68	2 (2.9)	66 (97.1)	38	7 (18.4)	31 (81.6)
	Mertolenga	41	0 (0.0)	41 (100.0)	115	21 (18.3)	94 (81.7)	1	0 (0.0)	1 (100.0)
	**Total**	65	1 (1.5)	64 (98.5)	183	23 (12.6)	160 (87.4)	39	7 (17.9)	32 (82.1)
**Portalegre**	Alentejana	28	0 (0.0)	28 (100.0)	149	9 (6.0)	140 (94.0)	25	3 (12.0)	22 (88.0)
	Mertolenga	60	0 (0.0)	60 (100.0)	22	7 (31.8	15 (68.2)	26	7 (26.9)	19 (73.1)
	**Total**	88	0 (0.0)	88 (100.0)	171	16 (9.4)	155 (90.6)	51	10 (19.6)	41 (80.4)
**Santarém**	Alentejana	0	0 (0.0)	0 (0.0)	6	0 (0.0)	6 (100.0)	0	0 (0.0)	0 (0.0)
	Mertolenga	1	0 (0.0)	1 (100.0)	27	2 (7.4)	25 (92.6)	9	2 (22.2)	7 (77.8)
	**Total**	1	0 (0.0)	1 (100.0)	33	2 (6.1)	31 (93.9)	9	2 (22.2)	7 (77.8)
**Setúbal**	Alentejana	0	0 (0.0)	0 (0.0)	7	0 (0.0)	7 (100.0)	8	1 (12.5)	7 (87.5)
	Mertolenga	0	0 (0.0)	0 (0.0)	0	0 (0.0)	0 (0.0)	0	0 (0.0)	0 (0.0)
	**Total**	0	0 (0.0)	0 (0.0)	7	0 (0.0)	7 (100.0)	8	1 (12.5)	7 (87.5)
**Total**		185	1 (0.5)	184 (99.5)	536	69 (12.9)	467 (87.1)	122	21 (17.2)	101 (82.8)

**Table 3:** *T. annulata* prevalence in animals belonging to farms classified according to NUTS III, their size (number of animals), the presence of dogs, the presence of other animals, the use of parasiticides and according to the month or season of the year in which the sample was collected. % prevalence is shown in parentheses.

		Alentejana Breed			Mertolenga Breed		
Parameter		Number of Samples	*T. annulata* Positive Samples (%)	*T. annulata* Negative Samples (%)	Number of Samples	*T. annulata* Positive Samples (%)	*T. annulata* Negative Samples (%)
**NUTS III**	Lezíria do Tejo	6	0 (0.0)	6 (100.0)	37	4 (10.8)	33 (89.2)
	Alto Alentejo	190	12 (6.3)	178 (93.7)	107	14 (13.1)	93 (86.9)
	Alentejo Central	142	10 (7.0)	132 (93.0)	158	21 (13.3)	137 (86.7)
	Baixo Alentejo	82	8 (9.8)	74 (90.2)	121	22 (18.2)	99 (81.8)
**Month of sampling**	Hot month	197	20 (10.2)	177 (89.8)	160	14 (8.8)	146 (91.3)
	Cold month	223	10 (4.5)	213 (95.5)	263	47 (17.9)	216 (82.1)
**Season**	Spring	66	9 (13.6)	57 (86.4)	116	1 (0.9)	115 (99.1)
	Summer	19	8 (42.1)	11 (57.9)	32	3 (9.4)	29 (90.6)
	Autumn	105	5 (4.8)	100 (95.2)	130	24 (18.5)	106 (81.5)
	Winter	230	8 (3.5)	222 (96.5)	145	33 (22.8)	112 (73.1)
**Number of animals on the farm**	<100 animals	47	5 (10.6)	42 (89.4)	70	19 (27.1)	51 (72.9)
	≥100 and <500 animals	307	21 (6.8)	286 (93.2)	327	37 (11.3)	290 (88.7)
	≥500 animals	66	4 (6.0)	62 (93.9)	26	5 (19.2)	21 (80.8)
**Presence of dogs**	Yes	260	22 (8.5)	238 (91.5)	340	49 (14.4)	291 (85.6)
	No	160	8 (5.0)	152 (95.0)	83	12 (14.5)	71 (85.5)
**Presence of other animals**	Yes	98	2 (2.0)	96 (98.0)	145	15 (10.3)	130 (89.6)
	No	322	28 (8.7)	294 (91.3)	278	46 (16.5)	232 (83.5)
**Use of ectoparasiticides**	Don’t know	0	0 (0.0)	0 (0.0)	6	2 (33.3)	4 (66.7)
	Not dewormed	0	0 (0.0)	0 (0.0)	11	3 (27.7)	8 (72.7)
	Ivermectin	355	29 (8.2)	326 (91.8)	195	41 (21.0)	154 (79.0)
	Ivermectin + Deltamethrin	0	0 (0.0)	0 (0.0)	79	3 (3.8)	76 (96.2)
	Ivermectin + Deltamethrin + Moxidectin	0	0 (0.0)	0 (0.0)	12	0 (0.0)	12 (100.0)
	Moxidectin	0	0 (0.0)	0 (0.0)	109	1 (0.9)	108 (99.1)
	Moxidectin + Cypermethrin	0	0 (0.0)	0 (0.0)	11	11 (100.0)	0 (0.0)
	Doramectin	59	1 (1.7)	58 (98.3)	0	0 (0.0)	0 (0.0)
	Deltamethrin	6	0 (0.0)	6 (100.0)	0	0 (0.0)	0 (0.0)

## Data Availability

Not applicable.
